# Engagement of Amyloid‐Targeting Antibodies on Native Human and Murine Cerebral Amyloid Angiopathy CAA Containing Vessels

**DOI:** 10.1002/alz70861_108893

**Published:** 2025-12-23

**Authors:** John A. Tzaferis, Justin T. Hole, Deon Turner, Ronald B. DeMattos

**Affiliations:** ^1^ Eli Lilly and Company, Indianapolis, IN USA

## Abstract

**Background:**

Amyloid‐related imaging abnormalities (ARIA) are the leading adverse event for amyloid‐targeting immunotherapies. The presence of CAA is a robust risk factor for ARIA development, and one hypothesis suggests that amyloid‐targeting antibody binding to CAA initiates the vascular insult. Therefore, we examined the ability of various clinical antibodies to engage CAA on native human and transgenic cerebral vessels.

**Method:**

**Aβ Characterization**:

Cerebral microvessels were isolated from 30‐month PDAPP or AD cortex using dextran solutions with centrifugation. Transgenic and human vessels were denatured in formic acid, and Aβ species were determined using acid urea gels (Aβ_x‐40_, Aβ_x‐42_, Aβ_p3‐x_).

**In‐Vitro and In‐Vivo Binding**:

Pooled native vessels were isolated from AD cortex and incubated with biotinylated human antibodies or their murine surrogates at multiple concentrations for 1‐hr. The human antibodies investigated were donanemab, lecanemab, gantenerumab, and bapineuzumab. Vessels were washed with PBS to remove any non‐bound antibody and subsequently denatured. The amount of antibody bound to CAA vessels was determined by sandwich ELISA and subsequently normalized to total Aβ_40_. For in‐vivo engagement, biotinylated mE8c (surrogate for donanemab) or 3D6 (surrogate for bapineuzumab) were injected intravenously into 22‐month transgenic mice and after 72 hours, animals were perfused, vessels isolated, and IgG and Aβ levels quantified by ELISA.

**Result:**

Aβ Characterization of PDAPP or human isolated vessels demonstrated an enrichment of Aβ_40_ and presence of low levels of Aβ_p3‐x_. ELISA results from the in‐vitro CAA binding assay demonstrated dose‐dependent CAA engagement for each antibody, with gantenerumab having the highest levels of binding, followed by similar engagement for bapineuzumab/lecanemab and donanemab having the least. The in‐vivo studies confirmed high CAA target engagement for the bapineuzumab surrogate as compared to negligible binding for donanemab or control IgG in aged transgenic mice.

**Conclusion:**

Characterization of Aβ peptides in isolated cerebral vessels confirmed a predominance of Aβ_40_ and low levels of truncated Aβ_p3‐x_. Higher levels of CAA target engagement were observed for amyloid‐targeting antibodies that bind deposited full‐length Aβ (gantenerumab, lecanemab, bapinezumab) as opposed to truncated and pyro‐glu modified Aβ (donanemab). These results suggest factors other than ability to bind CAA are important for the manifestation of ARIA.

